# Effects of different oral intake management during labor on maternal and neonatal outcomes: A protocol for a systematic review and network meta-analysis

**DOI:** 10.1371/journal.pone.0354489

**Published:** 2026-07-24

**Authors:** Zheng Nian, Yonghong Wang, Chuanya Huang, Xin Chen

**Affiliations:** 1 Department of Obstetrics Nursing, West China Second University Hospital, Sichuan University, Chengdu, Sichuan, China; 2 Key Laboratory of Birth Defects and Related Diseases of Women and Children (Sichuan University), Ministry of Education, Chengdu, Sichuan, China; UFRN: Universidade Federal do Rio Grande do Norte, BRAZIL

## Abstract

**Introduction:**

Fasting or restricting oral intake during labor may adversely affect maternal and neonatal outcomes. Although several interventions regarding oral intake during labor have been proposed, the optimal strategy for clinical practice remains controversial. This study aims to conduct a network meta-analysis to identify the most effective intervention for managing maternal oral intake during labor.

**Methods and analysis:**

A comprehensive search will be conducted in the following electronic databases: Web of Science, PubMed, Embase, Ovid, Cochrane Library, ClinicalTrials.gov, the WHO International Clinical Trials Registry Platform, the Chinese Biomedical Literature Database, China National Knowledge Infrastructure, Wanfang Database, and VIP Database, from database inception until January 2026. All randomized controlled trials (RCTs) evaluating oral intake interventions during labor will be included. The primary outcomes include vaginal delivery rate, operative vaginal delivery, caesarean section, 10‐minute Apgar score, and neonatal hypoglycemia. Secondary outcomes include maternal and neonatal outcomes. Maternal outcomes include duration of labor, ketoacidosis, maternal hypoglycemia, postpartum hemorrhage, vomiting rate, Mendelson’s syndrome, maternal thirst and hunger sensations, and maternal satisfaction. Neonatal outcomes include fetal distress, neonatal jaundice, umbilical cord blood pH < 7.2, and admission to the neonatal intensive care unit (NICU). Study selection, data extraction, and quality assessment will be conducted independently by two reviewers, and disagreements will be resolved through discussion with a third reviewer. Meta-analysis will be performed using RevMan 5.4 where appropriate; otherwise, subgroup analyses or descriptive analyses will be conducted.

**Ethics and dissemination:**

This review will not involve individual patient data and therefore does not require ethical approval. The findings of this systematic review and network meta-analysis will be disseminated through publication in a peer-reviewed journal and presentation at relevant academic conferences.

**PROSPERO registration number:**

CRD42025630953.

## Introduction

Vaginal delivery can be a prolonged process, with labor often lasting more than 10 hours, particularly among first-time mothers [1]. In 1946, Mendelson reported 66 cases of pulmonary aspiration during general anesthesia in obstetric patients, including two fatalities, which led to the routine practice of fasting during labor [2]. However, with advances in modern obstetric anesthesia, the use of general anesthesia during labor has become rare. Previous studies have reported that fewer than 1.5% of women in labor receive general anesthesia [3]. Even when general anesthesia is administered, the incidence of pulmonary aspiration remains very low, ranging from approximately 1 in 900 to 1 in 10,000 cases [4–6].

In addition, fasting during labor has been associated with reduced maternal comfort, lower satisfaction, diminished sense of control, and increased anxiety and stress [7–9]. Restricting oral intake may also led to inadequate energy, prolonged labor, and an increased likelihood of operative vaginal delivery [10,11]. Given the advances in obstetric practice and anesthesia techniques, as well as increasing attention to maternal preferences and childbirth experience, it is important to evaluate oral intake management strategies during labor to identify approaches that may benefit both mothers and newborns [12,13].

Oral intake management practices during labor vary considerably across countries and clinical guidelines. The 2016 Practice Guidelines for Obstetric Anesthesia issued by the American Society of Anesthesiologists recommend that women in labor may consume moderate amounts of clear liquids while avoiding solid foods [14]. In contrast, the World Health Organization (WHO) recommended that low-risk women be allowed to eat and drink according to their preferences during labor [15]. Similarly, the 2022 Queensland Clinical Guideline for Normal Birth encourages women to eat and drink as desired during labor [16].

Various oral intake strategies during labor have been investigated, including fasting, ice chips, sports drinks, high‐carbohydrate or isotonic beverages, high‐energy liquid diets, and individualized oral intake regimens. Previous meta-analyses have mainly focused on comparisons between two interventions, such as oral carbohydrate supplementation versus placebo or standard care [17], or restricted versus unrestricted oral intake [18]. However, direct comparisons among multiple oral intake strategies remain limited, and the optimal approach for improving maternal and neonatal outcomes remains unclear.

Network meta‐analysis (NMA) enables the simultaneous comparison of three or more interventions by integrating both direct and indirect evidence within a single analytical framework [19]. To our knowledge, no previous NMA has comprehensively compared different oral intake strategies during labor to evaluate their relative effects on maternal and neonatal outcomes. Therefore, conducting an NMA may help identify the most effective oral intake strategy and provide evidence to support clinical decision-making.

This study aims to systematically synthesize the available evidence on oral intake management during labor using NMA and to provide comprehensive evidence for optimizing nutritional management strategies for women during labor.

## Methods and analysis

This protocol was developed in accordance with the Preferred Reporting Items for Systematic Review and Meta-Analysis Protocols (PRISMA-P) statement and the Preferred Reporting Items for Systematic Reviews and Meta-Analyses extension for Network Meta-Analyses (PRISMA-NMA) guidelines [20,21]. The PRISMA-P checklist is provided in S1 File, and the reporting of the final review will follow PRISMA-NMA recommendations. The protocol has been registered in the International Prospective Register of Systematic Reviews (PROSPERO; registration number: CRD42025630953). The review has not yet commenced. Data collection is scheduled to begin in January 2026, and the study is expected to be completed within one year.

## Eligibility criteria

### Types of studies

We will include randomized controlled trials (RCTs), regardless of language or publication date. Only full-text published studies will be included. Conference abstracts, unpublished studies, and ongoing trials without available full data will be excluded.

### Participants

Women with a singleton cephalic pregnancy at ≥37 weeks of gestation who are admitted for planned vaginal birth.

### Interventions

Any structured oral intake intervention during labor will be considered. Interventions will be grouped into predefined categories according to the level of restriction and type of permitted intake. Examples include fasting during labor, restriction of oral intake (e.g., ice chips or minimal fluids), clear fluids (e.g., water or tea), carbohydrate-containing or isotonic beverages, high-energy or nutritional liquid diets, specific oral food regimens, and unrestricted oral intake.

### Outcome measures

Outcomes were selected based on their clinical relevance to maternal and neonatal safety, labor progress, metabolic status, and childbirth experience. Outcomes will be classified as maternal and neonatal outcomes.

#### Primary outcomes.

**Maternal outcomes:**

- Vaginal delivery rate
- Operative vaginal delivery
- Caesarean section

**Neonatal outcomes:**

- 10-minute Apgar score
- Neonatal hypoglycemia

The primary outcomes were selected because they directly reflect maternal and neonatal safety and the effectiveness of oral intake management during labor.

#### Secondary outcomes.

Secondary outcomes were selected to provide a comprehensive assessment of the safety, metabolic effects, and maternal experience associated with oral intake during labor, including rare but clinically important adverse events such as Mendelson’s syndrome.

**Maternal outcomes:**

- Duration of labor
- Ketoacidosis
- Maternal hypoglycemia
- Postpartum hemorrhage (PPH), commonly defined as cumulative blood loss ≥500 mL within 24 hours after vaginal delivery [22]
- Vomiting rate (defined as the proportion of women experiencing vomiting during labor, as reported in the original studies)
- Mendelson’s syndrome
- Thirst and hunger sensations
- Maternal satisfaction

Thirst and hunger sensations reflect maternal physical discomfort during labor, whereas maternal satisfaction reflects the overall childbirth experience.

**Neonatal outcomes:**

- Fetal distress
- Neonatal jaundice
- Umbilical Cord blood pH < 7.2
- Admission to the neonatal intensive care unit (NICU)

Outcome definitions and assessment time points will be extracted as reported in the original studies.

### Exclusion criteria

Studies will be excluded if they meet any of the following criteria: (1) non-randomized controlled trials, including cohort studies, cross-sectional studies, case-control studies, case reports, editorials, letters, conference abstracts, animal studies, reviews, and meta-analyses; (2) duplicate or redundant publications; and (3) studies with insufficient data or unsuccessful attempts to contact corresponding authors for missing information.

### Information sources and search strategy

A comprehensive literature search will be conducted in electronic databases including Web of Science, PubMed, Embase, Ovid, Cochrane Library, ClinicalTrials.gov, WHO International Clinical Trials Registry Platform, the Chinese Biomedical Literature Database, China National Knowledge Infrastructure (CNKI), Wanfang Database, and VIP Database. All records from database inception to January 2026 will be retrieved without language restrictions. The search strategy will be developed using Medical Subject Headings (MeSH) terms and relevant free-text keywords related to oral intake management during labor, maternal and neonatal outcomes, and randomized controlled trials. Equivalent strategies will be adapted for each database to ensure consistency and comprehensiveness. The detailed PubMed search strategy is presented in S1 Table.

### Study selection and data extraction

All retrieved studies will be organized and duplicates removed using EndNote 20. Two independent reviewers will screen titles and abstracts to determine study eligibility for inclusion in the systematic review and network meta‐analysis. When abstracts lack sufficient detail, the full text will be reviewed. Studies that do not meet the inclusion criteria will be excluded, and the reasons for exclusion will be documented. Disagreements between reviewers will be resolved by a third reviewer. The study selection process will be summarized in a PRISMA flow diagram [20].

Two reviewers will independently extract data using a specially created, standardized data extraction form. This form was designed by the research team for this review and tested on a sample of two to three included studies to guarantee accuracy and thoroughness. The form will capture data on:

- Study characteristics: first author, publication year, country, study design, settings, sample size, follow-up duration.
- Participant characteristics: inclusion and exclusion criteria, maternal age, gestational age, parity.
- Interventions and comparators: a detailed description of the oral intake management strategies in each arm (e.g., type and amount of food/fluid allowed, timing, protocol), including the specific brand and composition of any nutritional solutions.
- Outcomes: for both primary and secondary outcomes, we will extract the raw data necessary for meta-analysis (e.g., number of events and totals for dichotomous outcomes; means, standard deviations, and sample sizes for continuous outcomes).
- Data for network meta-analysis: we will extract the precise comparisons made between intervention arms to construct the network geometry.
- Other relevant data: information required for the ‘Risk of bias’ assessment and funding sources.

Following a cross-check of the extracted data, any differences will be discussed or, if required, decided by a third reviewer. To maintain data integrity, only full-text articles will be included. We will get in touch with the original studies’ corresponding authors to ask for more information if any important elements are omitted or unclear.

For data management and analysis, one reviewer will enter the data into RevMan 5.4 [23]. The accuracy of the data entry against the original extraction forms will be independently verified by a second reviewer to minimize transcription errors. The final RevMan file will serve as the primary dataset for all subsequent syntheses.

### Dealing with missing data

Following the methodology outlined in Chapter 10 of the Cochrane Handbook for Systematic Reviews of Interventions, we will deal with missing data [24]. Missing data will be addressed by:

- Recording incomplete outcome data and analyzing the available data based on the intention-to-treat (ITT) principle.
- Contacting original trial investigators for additional information when data are insufficient or missing.
- Conducting sensitivity analyses if missing data are deemed likely to introduce significant bias.
- Discussing the potential impact of missing data on the overall findings of the review in the Discussion section.

### Risk of bias assessment

Two researchers will independently assess the risk of bias of each included study using the revised Cochrane Risk of Bias 2 (RoB 2) tool for randomized trials, as described in the Cochrane Handbook for Systematic Reviews of Interventions (version 6.5.0) [19]. Any disagreements arising during the assessment process will be resolved through discussion with a third reviewer. The RoB 2 tool evaluates five domains: randomization process, deviations from intended interventions, missing outcome data, measurement of the outcome, and selection of the reported result. Each domain includes a series of signaling questions with five response options: “Yes”, “Probably yes”, “Probably no”, “No”, and “No information”. Based on the responses, the risk of bias for each domain will be judged as “low risk”, “some concerns”, or “high risk”.

An overall risk-of-bias judgment will then be assigned for each study. Studies with low risk in all domains will be considered at overall low risk of bias. Studies with some concerns in at least one domain, but without any high-risk domains, will be judged as having some concerns overall. Studies with at least one domain rated as high risk will be considered at overall high risk of bias [25].

The RoB 2 Excel tool will be used to manage and document the risk-of-bias assessments. All assessment data will be made publicly available as supplementary material on the Open Science Framework platform.

Studies will not be excluded solely on the basis of risk of bias. However, sensitivity analyses may be conducted by excluding studies judged to be at high risk of bias, where sufficient data are available, to evaluate the robustness of the findings. The results of the risk of bias assessment will be considered when interpreting the findings and assessing the certainty of evidence.

### Data synthesis

For dichotomous outcomes (e.g., vaginal delivery rate, cesarean section, and operative vaginal delivery), treatment effects will be expressed as risk ratios (RRs) with 95% confidence intervals (CIs). For continuous outcomes (e.g., duration of labor and postpartum blood loss volume), treatment effects will be expressed as mean differences (MDs) with 95% CIs. Standardized mean differences (SMDs) will be used when the same outcome is assessed using different measurement scales.

Pairwise meta-analyses will be conducted in RevMan 5.4 using a random-effects model when appropriate. Heterogeneity will be assessed using the I^2^ statistic and Chi-square test. When substantial heterogeneity (I^2^ > 50%) is detected, potential sources will be explored through subgroup analyses or sensitivity analyses.

### Intervention classification

To reduce clinical heterogeneity, interventions will be grouped into predefined nodes according to the level of oral intake restriction and type of permitted intake during labor.

The predefined nodes include fasting, restricted intake, clear fluids, carbohydrate-containing beverages, high-energy or nutritional intake, and unrestricted oral intake.

When a study includes more than one oral intake strategy, it will be classified according to the primary or intended dietary policy reported by the original study. Any uncertainty in classification will be resolved through discussion among the review team.

### Network meta-analysis

A Bayesian random-effects network meta-analysis will be conducted using R software to integrate direct and indirect evidence across multiple interventions [26]. The transitivity assumption will be assessed by comparing potential effect modifiers across studies, including maternal characteristics, gestational age, labor management practices, and intervention delivery settings.

Consistency between direct and indirect evidence will be evaluated using node-splitting analyses when applicable [27]. Inconsistency will be considered present when significant differences are detected between direct and indirect estimates. Multi-arm trials will be incorporated into the network model while accounting for correlations between effect sizes.

Model convergence will be assessed using standard diagnostic tools, including trace plots and the Potential Scale Reduction Factor (PSRF), with values approaching 1 indicating adequate convergence. Non-informative prior distributions will be used for all model parameters.

The relative ranking of interventions will be estimated using the surface under the cumulative ranking curve (SUCRA). SUCRA values will be interpreted alongside effect estimates and their 95% confidence intervals to avoid over-reliance on ranking alone. Network plots will be generated to visualize the geometry of the treatment comparisons.

### Assessment of heterogeneity

Clinical and methodological heterogeneity will be assessed by examining similarities in participant characteristics, interventions, outcomes, and study designs. Forest plots from pairwise meta‐analyses will be used for visual assessment. Statistical heterogeneity will be quantified using the I^2^ statistic (with I^2^ > 50% suggesting substantial heterogeneity), and between-study variance (τ^2^) will be considered where appropriate. Strategies to address heterogeneity will include [24]:

- Verifying data accuracy
- Opting not to perform a meta-analysis when heterogeneity is excessive
- Conducting subgroup analyses or meta‐regression to explore heterogeneity
- Utilizing a random‐effects model
- Reconsidering the choice of effect measure
- Excluding outlier studies

### Assessment of reporting biases

Reporting biases, which may be influenced by the nature and direction of study results [28], will be assessed. If sufficient studies are available, funnel plots and comparison-adjusted funnel plots will be constructed using STATA 18.0 to assess potential publication bias and small-study effects in the network meta-analysis.

### Sensitivity analysis

Sensitivity analyses will be conducted using leave-one-out analyses and subgroup analyses to evaluate the robustness and stability of the study results.

### Assessing the certainty of evidence

The Grading of Recommendations Assessment, Development and Evaluation (GRADE) system will be used to evaluate the quality of evidence from both pairwise and network meta‐analyses. Five key domains will be considered: study limitations, indirectness, inconsistency, imprecision, publication bias. Based on these domains, the certainty of evidence will be categorized as high, moderate, low, or very low [29].

### Ethics and dissemination

This study will not involve data collection from human participants, so it will not require ethical approval. We will publish our findings in a peer-reviewed journal and may also present them at conferences.

### Strengths and limitations of this study

- This study will conduct a network meta-analysis protocol to compare various oral intake managements during labor to determine their relative efficacy and safety on maternal and newborn outcomes.
- The study may provide reliable evidence-based medicine for the management of maternal oral intake during delivery.
- This protocol will strictly adhere to the PRISMA guidelines for systematic reviews and meta-analyses.
- The inclusion of various interventions may lead to data heterogeneity and potential biases in interpretation.

## Supporting information

S1 File PRISMA-P checklist. (DOCX)

S1 Table Detailed search strategy for PubMed. (DOCX)
