# Supplementary material for: Effects of different oral intake management during labor on maternal and neonatal outcomes: A protocol for a systematic review and network meta-analysis
Source: PLoS One. 2026 Jul 24;21(7):e0354489. doi: 10.1371/journal.pone.0354489 (PMC13399317; doi:10.1371/journal.pone.0354489)
Supplement: S1 Table — (DOCX) [file pone.0354489.s002.docx]

**Table S1. Detailed search strategy for PubMed.**

| Order | Strategy |
| --- | --- |
| #1 | "oral intake" OR "food intake" OR "fluid intake" OR "eating" OR "drinking" OR "feeding" |
| #2 | "labour" OR "labor" OR "childbirth" OR "intrapartum" |
| #3 | #1 AND #2 |
| #4 | "intrapartum nutrition" OR "intrapartum feeding" OR "eating in labour" OR "eating in labor" OR "drinking during labour" OR "drinking during labor" |
| #5 | "fasting in labour" OR "fasting in labor" OR "food restriction" OR "fluid restriction" OR "nil by mouth" OR "NPO" |
| #6 | #2 AND #5 |
| #7 | "clear fluid*" OR "carbohydrate solution" OR "carbohydrate drink" OR "energy drink" OR "light diet" OR "oral hydration" |
| #8 | #2 AND #7 |
| #9 | "diet" OR "nutrition" OR "nutritional support" OR "dietary intake" |
| #10 | #2 AND #9 |
| #11 | "Fasting"[Mesh] |
| #12 | "Labor, Obstetric"[Mesh] OR "Parturition"[Mesh] |
| #13 | #11 AND #12 |
| #14 | #3 OR #4 OR #6 OR #8 OR #10 OR #13 |
| #15 | “Randomized Controlled Trials”[Mesh] OR “Clinical Trials, Randomized” OR “Trials, Randomized Clinical” OR “Controlled Clinical Trials, Randomized” OR “randomized” |
| #16 | #14 AND #15 |
