# Supplementary material for: Effects of different oral intake management during labor on maternal and neonatal outcomes: A protocol for a systematic review and network meta-analysis
Source: PLoS One. 2026 Jul 24;21(7):e0354489. doi: 10.1371/journal.pone.0354489 (PMC13399317; doi:10.1371/journal.pone.0354489)
Supplement: Attachment — Submitted filename: PLOS One Response_to_Reviewers.docx [file pone.0354489.s003.docx]

Jan 5, 2026

*PLOS ONE*

Dear Editor-in-Chief,

We are pleased to re-submit our revised manuscript titled “**Effect of different oral intake managements during labor on maternal and newborn outcomes: a protocol of systematic review and network meta-analysis.”** (Manuscript ID: **PONE-D-25-66860**) for further consideration in your esteemed journal.

We sincerely appreciate the time and effort invested by you in evaluating our work. Your constructive feedback and insightful suggestions have significantly strengthened the quality and clarity of our manuscript.

We have revised the manuscript to fully address all the valuable recommendations provided. All changes are highlighted in red font for ease of reference. Below we provided a point-by-point response to the reviewers’ comments.

Thank you once again for your guidance throughout the review process. We look forward to your feedback.

Sincerely,

Xin Chen

Key Laboratory of Birth Defects and Related Diseases of Women and Children (Sichuan University), Ministry of Education, Chengdu, Sichuan, China

E-mail: chenxin123@stu.scu.edu.cn

**Effect of different oral intake managements during labor on maternal and newborn outcomes: a protocol of systematic review and network meta-analysis**

**Manuscript ID: PONE-D-25-66860**

**Response to the Editor**

**Comment 1: We note that your study timeline is missing from your manuscript. Specifically, it is unclear whether your study has begun thus if the manuscript is submitted as the appropriate article type. If your study has already begun, it may be considered as a Study Protocol (https://journals.plos.org/plosone/s/what-we-publish/#loc-study-protocols) rather than a Registered Report Protocol (https://journals.plos.org/plosone/s/other-article-types#loc-registered-reports).**

Response: We sincerely appreciate your comment regarding the stage of our research. This is an important point of clarification, and we are grateful for the opportunity to provide more details. We would like to clarify that this systematic review and network meta-analysis is strictly at the protocol stage. To ensure transparency and reduce bias, we have prospectively registered the review on PROSPERO (Registration number: CRD42025630953). To date, we have not commenced any study screening, data extraction, or statistical analysis. All future work will be conducted in strict accordance with the methods outlined in this submitted protocol. So, we submit the manuscript according to the "Study Protocol".

We are happy to proceed as advised by your journal's policy. We kindly ask for your guidance on the most suitable path forward:1. To continue the review process as a "Study Protocol" manuscript, or 2. If supported by the journal, to consider it as a submission under a "Registered Report" track.

Please let us know your preferred course of action. We are fully prepared to revise the manuscript according to any specific formatting or content requirements for its designated article type, based on your feedback.

**Comment 2: Please clarify if your study has begun, when the final search will be conducted for your network meta-analysis, and when your study will be complete. Please include this information in your Methods section.**

Response: Thank you for your suggestion. The study has not yet started. It is scheduled to start in January 2026 and is planned to be completed within a year. We have revised in our Methods Section ( Methods and analysis, page3, highlighted in red).
