# Supplementary material for: Effects of different oral intake management during labor on maternal and neonatal outcomes: A protocol for a systematic review and network meta-analysis
Source: PLoS One. 2026 Jul 24;21(7):e0354489. doi: 10.1371/journal.pone.0354489 (PMC13399317; doi:10.1371/journal.pone.0354489)
Supplement: Attachment — Submitted filename: PLOS_One__Response_to_Reviewers_auresp_1.docx [file pone.0354489.s004.docx]

June 8, 2026

*PLOS ONE*

Dear Editor-in-Chief,

We are pleased to re-submit our revised manuscript titled “**Effect of different oral intake managements during labor on maternal and newborn outcomes: a protocol of systematic review and network meta-analysis.”** (Manuscript ID: **PONE-D-25-66860**) for further consideration in your esteemed journal.

We sincerely appreciate the time and effort invested by you in evaluating our work. Your constructive feedback and insightful suggestions have significantly strengthened the quality and clarity of our manuscript.

We have revised the manuscript to fully address all the valuable recommendations provided. All changes are highlighted in red font for ease of reference. Below we provided a point-by-point response to the reviewers’ comments.

Thank you once again for your guidance throughout the review process. We look forward to your feedback.

Sincerely,

Xin Chen

Key Laboratory of Birth Defects and Related Diseases of Women and Children (Sichuan University), Ministry of Education, Chengdu, Sichuan, China

E-mail: chenxin123@stu.scu.edu.cn

**Effect of different oral intake managements during labor on maternal and newborn outcomes: a protocol of systematic review and network meta-analysis**

**Manuscript ID: PONE-D-25-66860**

**Response to the Editor**

**Comment 1: Please ensure that your manuscript meets PLOS ONE's style requirements, including those for file naming. The PLOS ONE style templates can be found at**

**https://journals.plos.org/plosone/s/file?id=wjVg/PLOSOne_formatting_sample_main_body.pdf and**

[**https://journals.plos.org/plosone/s/file?id=ba62/PLOSOne_formatting_sample_title_authors_affiliations.pdf**](https://track.editorialmanager.com/CL0/https:%2F%2Fjournals.plos.org%2Fplosone%2Fs%2Ffile%3Fid=ba62%2FPLOSOne_formatting_sample_title_authors_affiliations.pdf/1/010f019c9cb802cb-f9f70348-7eb6-4e0d-ba37-6c6cee628f0a-000000/JRyyfndoOXUNNEPOceWbyLlJWvBKuQtwrTbLqF1wGdk=252)

Response: Thank you for your helpful comments. We have carefully revised the manuscript to ensure that it complies with the PLOS ONE formatting requirements. Specifically, we have adjusted the title to sentence case format, standardized the author names and affiliations, and revised the manuscript according to the PLOS ONE templates. All formatting issues have been checked and corrected throughout the manuscript. (page1, title page).

**Comment 2: Please provide a complete Data Availability Statement in the submission form, ensuring you include all necessary access information or a reason for why you are unable to make your data freely accessible. If your research concerns only data provided within your submission, please write "All data are in the manuscript and/or supporting information files" as your Data Availability Statement.**

Response: Thank you for your comment. As this study is a protocol for a systematic review and network meta-analysis, no datasets were generated or analyzed. We have provided a complete Data Availability Statement in both the manuscript and the submission system, clarifying that all data will be obtained from published studies. (page 2, Data Availability Statement section)

**Comment 3：When completing the data availability statement of the submission form, you indicated that you will make your data available on acceptance. We strongly recommend all authors decide on a data sharing plan before acceptance, as the process can be lengthy and hold up publication timelines. Please note that, though access restrictions are acceptable now, your entire data will need to be made freely accessible if your manuscript is accepted for publication. This policy applies to all data except where public deposition would breach compliance with the protocol approved by your research ethics board. If you are unable to adhere to our open data policy, please kindly revise your statement to explain your reasoning and we will seek the editor's input on an exemption. Please be assured that, once you have provided your new statement, the assessment of your exemption will not hold up the peer review process.**

Response: Thank you for your comment. We have revised the data availability statement in the submission form accordingly. As this study is a protocol for a systematic review and network meta-analysis, no datasets were generated or analyzed at this stage. We have therefore clarified that all data will be obtained from previously published studies, and have removed the statement indicating that data would be made available upon acceptance. (page 2, Data Availability Statement section).

**Comment 4：Please include captions for your Supporting Information files at the end of your manuscript, and update any in-text citations to match accordingly. Please see our Supporting Information guidelines for more information:** [**http://journals.plos.org/plosone/s/supporting-information**](http://track.editorialmanager.com/CL0/http:%2F%2Fjournals.plos.org%2Fplosone%2Fs%2Fsupporting-information/1/010f019c9cb802cb-f9f70348-7eb6-4e0d-ba37-6c6cee628f0a-000000/t1hMDMj9gw_KNPMVOHm3zW1lbzdw9qNy7yI5zOJVyQk=252)**.**

Response: We have added captions for all Supporting Information files at the end of the manuscript and updated the in-text citations to ensure consistency with the journal’s requirements. (page 9, Supporting information section).

**Comment 5：If the reviewer comments include a recommendation to cite specific previously published works, please review and evaluate these publications to determine whether they are relevant and should be cited. There is no requirement to cite these works unless the editor has indicated otherwise.**

Response：Thank you for your guidance and will take it into consideration.

**Response to the reviewer 1**

**Comment 1：The proposal needs language refinements throughout the protocol for clarity.**

Response: Thank you for this valuable comment. We have carefully revised the language throughout the manuscript to improve clarity, readability, and academic expression. Specifically, grammatical structures, sentence flow, and terminology have been thoroughly refined. All revisions have been highlighted in the revised manuscript for ease of review.

**Comment 2：The abstract mentions "any other" secondary outcomes. Secondary outcomes need to be clearly stated and pre-specified to avoid bias.**

Response: Thank you for this comment. We have clearly predefined and fully listed all secondary outcomes in both the abstract and main text. All vague expressions have been removed, and no additional outcomes will be added. This revision improves transparency and consistency with the protocol. (page 4, Outcomes section).

**Comment 3：Eligibility criteria. How can you include unpublished RCTs?**

Response: Thank you for this important comment. We have revised the eligibility criteria to clarify that only full-text published randomized controlled trials will be included. Unpublished studies, conference abstracts, and ongoing trials without full texts will be excluded. This clarification improves the feasibility and consistency of the study design. (page 4, Types of studies section).

**Comment 4：Please specify how to assess "vomiting rate"**

Response: Thank you for this comment. Vomiting rate has been clarified in the secondary outcomes as reported in the original studies, and the revision has been marked in red. (page 4, Secondary Outcomes section).

**Comment 5：Outcomes of ‘sensations of thirst and hunger’ and ‘maternal satisfaction’ seems overlapping.**

Response: Thank you for this comment. We agree that these two outcomes may appear conceptually similar. We have clarified that “thirst and hunger sensations” refer to maternal subjective physical discomfort during labor, whereas “maternal satisfaction” reflects the overall evaluation of the birth experience. Both outcomes are retained as they measure different constructs. (page 4, Secondary Outcomes section).

**Comment 6：Protocol emphasizes the use of different statistical packages: RevMan, R software, STATA…. Explicitly state how, when and where those will be used.**

Response: Thank you for this comment. We have clarified the specific roles of the statistical software packages used in this review in the Data synthesis section. RevMan 5.4 will be used for pairwise meta-analysis and data management, R software for Bayesian network meta-analysis, and STATA 18.0 for network plotting and assessment of publication bias. The corresponding revisions have been highlighted in red. (pages 7-8, Data synthesis section, Network meta-analysis section and Assessment of reporting biases section).

**Response to the reviewer 2**

**Comment 1: Insufficient detail on network meta-analysis methodology: Important missing details include: Model specification (fixed vs random for NMA — only random mentioned generically),Prior distributions (Bayesian analysis requires specification), Convergence diagnostics (e.g., Gelman-Rubin, trace plots), Number of iterations / burn-in, Assessment of transitivity assumption, Assessment of consistency/inconsistency, node-splitting, Design-by-treatment interaction model, Handling of multi-arm trials, Ranking uncertainty interpretation. These are essential for reproducibility and credibility.**

Response: Thank you for this valuable comment. We have added a new Network meta-analysis section in the Data synthesis part of the revised manuscript to provide more detailed methodological descriptions as suggested. The corresponding revisions have been highlighted in red. (page 8, Network meta-analysis section).

**Comment 2: Intervention classification is unclear: The intervention category “oral intake management” is extremely broad. Examples listed include: Fasting, Clear fluids, Carbohydrate drinks, High-energy liquids, Individualized regimens, Unrestricted intake. Without predefined grouping rules, there is high risk of Clinical heterogeneity. The protocol should specify Planned intervention nodes, Criteria for merging/splitting nodes, Handling of mixed interventions (e.g., fluids + solids).**

Response: Thank you for this valuable comment. We agree that the original description of oral intake interventions was too broad and may lead to ambiguity in network meta-analysis. In the revised manuscript, we have specified predefined intervention nodes, clarified the criteria for grouping interventions, and described how mixed interventions will be handled. These revisions have been highlighted in red in the manuscript. (page 7, Intervention classification section).

**Comment 3: Outcome selection rationale is unclear. Primary outcomes include delivery mode and neonatal hypoglycemia. Some outcomes may be rare (e.g., Mendelson’s syndrome).Maternal satisfaction is listed as secondary but is highly relevant. The authors should Provide clinical reasoning, Define time points clearly, Specify measurement definitions (e.g., PPH threshold)**

Response: Thank you for this comment. We have revised the Outcome Measures section to provide the clinical rationale for outcome selection and to clarify key outcome definitions. We have also specified that outcome definitions and assessment time points will be extracted as reported in the original studies. In addition, we clarified the inclusion of rare but clinically important adverse events, such as Mendelson’s syndrome. All revisions have been highlighted in red. (page 4, Outcome measures section).

**Comment 4: Pairwise analysis plan is confusing. The protocol states: A pairwise Bayesian meta-analysis followed by a network meta-analysis.Typically, Pairwise meta-analysis is frequentist NMA can be Bayesian or frequentist, Clarification is needed.**

Response: Thank you for this helpful comment. We have clarified the statistical framework used for pairwise and network meta-analyses. Pairwise meta-analyses are now described separately in the Data Synthesis section and will be conducted in RevMan 5.4 using a random-effects model. The Bayesian random-effects network meta-analysis is described in a separate Network Meta-analysis section. The relevant revisions have been highlighted in red. (pages 7-8, Data synthesis section and Network meta-analysis section).

**Comment 5: Software inconsistency. The manuscript mentions RevMan 5.4, R software, STATA 18. This creates confusion regarding Primary analytic platform and Reproducibility pipeline. A clearer analytic workflow is needed.**

Response: Thank you for this comment. We have clarified the analytical workflow throughout the manuscript. RevMan 5.4 will be used for data management and pairwise meta-analyses, R software for Bayesian network meta-analysis, and STATA 18.0 for publication bias assessment. These functions are now specified in the corresponding sections and highlighted in red. (pages 7-8, Data synthesis section, Network meta-analysis section and Assessment of reporting biases section).

**Comment 6: Risk of bias synthesis not describe. While RoB2 is planned, the protocol does not state how bias will influence analysis, Sensitivity analyses, Exclusion of high-risk studies, and Meta-regression. This should be specified.**

Response: Thank you for this comment. We have revised the Risk of Bias Assessment section to clarify how risk of bias will be incorporated into the review. Sensitivity analyses excluding studies at high risk of bias may be conducted where appropriate, and the risk of bias assessment will be considered when interpreting the findings and assessing the certainty of evidence. The relevant revisions have been highlighted in red. (pages 6-7, Risk of bias assessment section).

**Response to Reviewer – Moderate concern**

**Comment 1: Search strategy may miss relevant studies. The PubMed search appears limited in synonyms for labor nutrition, Does not clearly include anesthesia-related terms that may capture relevant trials. A full reproducible search strategy for all databases should be included in supplementary materials**

Response: Thank you for this comment. We have clarified that a comprehensive literature search will be conducted across multiple databases using both Medical Subject Headings (MeSH) terms and free-text keywords. Equivalent search strategies are applied across all databases to ensure consistency and comprehensiveness. The detailed PubMed search strategy is provided in Table S2. The manuscript has been revised accordingly. (page 5, Information sources and search strategy section).

**Comment 2: Heterogeneity description is simplistic. Statement that “non-overlapping confidence intervals indicate heterogeneity” is incorrect or oversimplified. Heterogeneity assessment should rely on:I², τ² and Clinical judgment.**

Response: Thank you for this comment. We have revised the Assessment of Heterogeneity section by removing the statement regarding non-overlapping confidence intervals and clarifying that heterogeneity will be assessed using clinical and methodological judgment together with statistical measures, including the I² statistic and between-study variance (τ²), where appropriate. The relevant revisions have been highlighted in red. (page 8, Assessment of Heterogeneity section).

**Comment 3: Reporting bias assessment in NMA. Funnel plots alone are insufficient for NMA. Consider Comparison-adjusted funnel plots, Small-study effects analysis.**

Response: Thank you for this comment. We have revised the Assessment of Reporting Biases section to clarify that, where sufficient studies are available, both funnel plots and comparison-adjusted funnel plots will be used to assess potential publication bias and small-study effects in the network meta-analysis. The relevant revisions have been highlighted in red. (page 8, Assessment of Reporting Biases section).
