# Supplementary material for: Effects of different oral intake management during labor on maternal and neonatal outcomes: A protocol for a systematic review and network meta-analysis
Source: PLoS One. 2026 Jul 24;21(7):e0354489. doi: 10.1371/journal.pone.0354489 (PMC13399317; doi:10.1371/journal.pone.0354489)
Supplement: Peer review history

# Author comment: Author response to Decision Letter 0

**DOI:** 10.1371/journal.pone.0354489.r001

5 Jan 2026

Attachment Submitted filename: PLOS One Response_to_Reviewers.docx

# Peer review: Decision Letter 0

**DOI:** 10.1371/journal.pone.0354489.r002

**Authors:** Ricardo Ney Oliveira Cobucci

26 Feb 2026

PONE-D-25-66860

Effect of different oral intake managements during labor on maternal and newborn outcomes: a protocol of systematic review and network meta-analysis.

PLOS One

Dear Dr. Chen,

Thank you for submitting your manuscript to PLOS ONE. After careful consideration, we feel that it has merit but does not fully meet PLOS ONE’s publication criteria as it currently stands. Therefore, we invite you to submit a revised version of the manuscript that addresses the points raised during the review process.

Dear authors, as you can see, the reviewers have requested substantial revisions to your manuscript. We are certainly willing to reconsider a revised submission, but please know that this is not preliminary acceptance of your paper. When returning your revised manuscript, please be sure to include a point-by-point summary of the suggestions of the reviewers that specifies how and where in the text you have addressed the suggestions.

Please submit your revised manuscript by Apr 12 2026 11:59PM. If you will need more time than this to complete your revisions, please reply to this message or contact the journal office at plosone@plos.org. When you're ready to submit your revision, log on to https://www.editorialmanager.com/pone/ and select the 'Submissions Needing Revision' folder to locate your manuscript file.

Please include the following items when submitting your revised manuscript:

- A letter that responds to each point raised by the academic editor and reviewer(s). You should upload this letter as a separate file labeled 'Response to Reviewers'.
- A marked-up copy of your manuscript that highlights changes made to the original version. You should upload this as a separate file labeled 'Revised Manuscript with Track Changes'.
- An unmarked version of your revised paper without tracked changes. You should upload this as a separate file labeled 'Manuscript'.

If you would like to make changes to your financial disclosure, please include your updated statement in your cover letter. Guidelines for resubmitting your figure files are available below the reviewer comments at the end of this letter.

If applicable, we recommend that you deposit your laboratory protocols in protocols.io to enhance the reproducibility of your results. Protocols.io assigns your protocol its own identifier (DOI) so that it can be cited independently in the future. For instructions see: https://journals.plos.org/plosone/s/submission-guidelines#loc-laboratory-protocols. Additionally, PLOS ONE offers an option for publishing peer-reviewed Lab Protocol articles, which describe protocols hosted on protocols.io. Read more information on sharing protocols at https://plos.org/protocols?utm_medium=editorial-email&utm_source=authorletters&utm_campaign=protocols.

We look forward to receiving your revised manuscript.

Kind regards,

Ricardo Ney Oliveira Cobucci, Ph.D

Academic Editor

PLOS One

Journal Requirements:

When submitting your revision, we need you to address these additional requirements.

1. Please ensure that your manuscript meets PLOS ONE's style requirements, including those for file naming. The PLOS ONE style templates can be found at

https://journals.plos.org/plosone/s/file?id=wjVg/PLOSOne_formatting_sample_main_body.pdf and

https://journals.plos.org/plosone/s/file?id=ba62/PLOSOne_formatting_sample_title_authors_affiliations.pdf

2. Please provide a complete Data Availability Statement in the submission form, ensuring you include all necessary access information or a reason for why you are unable to make your data freely accessible. If your research concerns only data provided within your submission, please write "All data are in the manuscript and/or supporting information files" as your Data Availability Statement.

3. When completing the data availability statement of the submission form, you indicated that you will make your data available on acceptance. We strongly recommend all authors decide on a data sharing plan before acceptance, as the process can be lengthy and hold up publication timelines. Please note that, though access restrictions are acceptable now, your entire data will need to be made freely accessible if your manuscript is accepted for publication. This policy applies to all data except where public deposition would breach compliance with the protocol approved by your research ethics board. If you are unable to adhere to our open data policy, please kindly revise your statement to explain your reasoning and we will seek the editor's input on an exemption. Please be assured that, once you have provided your new statement, the assessment of your exemption will not hold up the peer review process.

4. Please include captions for your Supporting Information files at the end of your manuscript, and update any in-text citations to match accordingly. Please see our Supporting Information guidelines for more information: http://journals.plos.org/plosone/s/supporting-information.

5. If the reviewer comments include a recommendation to cite specific previously published works, please review and evaluate these publications to determine whether they are relevant and should be cited. There is no requirement to cite these works unless the editor has indicated otherwise.

[Note: HTML markup is below. Please do not edit.]

Reviewers' comments:

Reviewer's Responses to Questions

**Comments to the Author**

1. Does the manuscript provide a valid rationale for the proposed study, with clearly identified and justified research questions?

The research question outlined is expected to address a valid academic problem or topic and contribute to the base of knowledge in the field.

Reviewer #1: Yes

Reviewer #2: Yes

**********

2. Is the protocol technically sound and planned in a manner that will lead to a meaningful outcome and allow testing the stated hypotheses?

The manuscript should describe the methods in sufficient detail to prevent undisclosed flexibility in the experimental procedure or analysis pipeline, including sufficient outcome-neutral conditions (e.g. necessary controls, absence of floor or ceiling effects) to test the proposed hypotheses and a statistical power analysis where applicable. As there may be aspects of the methodology and analysis which can only be refined once the work is undertaken, authors should outline potential assumptions and explicitly describe what aspects of the proposed analyses, if any, are exploratory.

Reviewer #1: Yes

Reviewer #2: Partly

**********

3. Is the methodology feasible and described in sufficient detail to allow the work to be replicable?

Descriptions of methods and materials in the protocol should be reported in sufficient detail for another researcher to reproduce all experiments and analyses. The protocol should describe the appropriate controls, sample size calculations, and replication needed to ensure that the data are robust and reproducible.

Reviewer #1: Yes

Reviewer #2: Yes

**********

4. Have the authors described where all data underlying the findings will be made available when the study is complete?

The PLOS Data policy requires authors to make all data underlying the findings described in their manuscript fully available without restriction, with rare exception, at the time of publication. The data should be provided as part of the manuscript or its supporting information, or deposited to a public repository. For example, in addition to summary statistics, the data points behind means, medians and variance measures should be available. If there are restrictions on publicly sharing data—e.g. participant privacy or use of data from a third party—those must be specified.

Reviewer #1: Yes

Reviewer #2: Yes

**********

5. Is the manuscript presented in an intelligible fashion and written in standard English?

PLOS ONE does not copyedit accepted manuscripts, so the language in submitted articles must be clear, correct, and unambiguous. Any typographical or grammatical errors should be corrected at revision, so please note any specific errors here.

Reviewer #1: Yes

Reviewer #2: Yes

**********

6. Review Comments to the Author

Please use the space provided to explain your answers to the questions above and, if applicable, provide comments about issues authors must address before this protocol can be accepted for publication. You may also include additional comments for the author, including concerns about research or publication ethics.

You may also provide optional suggestions and comments to authors that they might find helpful in planning their study.

(Please upload your review as an attachment if it exceeds 20,000 characters)

Reviewer #1: I read the authors' proposal for a systematic review and meta analysis with great interest. The topic is important and timely for exploration. The proposal is over all well presented.

I have following suggestions and comments.

1.The proposal needs language refinements throughout the protocol for clarity.

2.The abstract mentions "any other" secondary outcomes. Secondary outcomes need to be clearly stated and pre-specified to avoid bias.

3.Eligibility criteria. How can you include unpublished RCTs?

4. Please specify how to assess "vomiting rate"

5.Outcomes of ‘sensations of thirst and hunger’ and ‘maternal satisfaction’ seems overlapping.

6.Protocol emphasizes the use of different statistical packages: RevMan, R software, STATA…. Explicitly state how, when and where those will be used.

I wish authors all the best in their endeavour.

Reviewer #2: 1. Insufficient detail on network meta-analysis methodology:

Important missing details include: Model specification (fixed vs random for NMA — only random mentioned generically),Prior distributions (Bayesian analysis requires specification), Convergence diagnostics (e.g., Gelman-Rubin, trace plots), Number of iterations / burn-in, Assessment of transitivity assumption, Assessment of consistency/inconsistency, node-splitting, Design-by-treatment interaction model, Handling of multi-arm trials, Ranking uncertainty interpretation.

These are essential for reproducibility and credibility.

2. Intervention classification is unclear

The intervention category “oral intake management” is extremely broad.

Examples listed include:Fasting,Clear fluids,Carbohydrate drinks,High-energy liquids,Individualized regimens,Unrestricted intake. Without predefined grouping rules, there is high risk of Clinical heterogeneity.

The protocol should specify Planned intervention nodes,Criteria for merging/splitting nodes, Handling of mixed interventions (e.g., fluids + solids).

3. Outcome selection rationale is unclear

Primary outcomes include delivery mode and neonatal hypoglycemia. Some outcomes may be rare (e.g., Mendelson’s syndrome).

Maternal satisfaction is listed as secondary but is highly relevant

The authors should Provide clinical reasoning, Define time points clearly, Specify measurement definitions (e.g., PPH threshold)

4. Pairwise analysis plan is confusing

The protocol states: A pairwise Bayesian meta-analysis followed by a network meta-analysis.

Typically, Pairwise meta-analysis is frequentist NMA can be Bayesian or frequentist, Clarification is needed.

5. Software inconsistency

The manuscript mentions RevMan 5.4, R software, . STATA 18

This creates confusion regarding Primary analytic platform and Reproducibility pipeline. A clearer analytic workflow is needed.

6. Risk of bias synthesis not described

While RoB2 is planned, the protocol does not state how bias will influence analysis, Sensitivity analyses, Exclusion of high-risk studies, and Meta-regression. This should be specified.

Moderate concerns

1. Search strategy may miss relevant studies

The PubMed search appears limited in synonyms for labor nutrition, Does not clearly include anesthesia-related terms that may capture relevant trials.

A full reproducible search strategy for all databases should be included in supplementary materials.

2. Heterogeneity description is simplistic

Statement that “non-overlapping confidence intervals indicate heterogeneity” is incorrect or oversimplified.

Heterogeneity assessment should rely on:I², τ² and Clinical judgment

3. Reporting bias assessment in NMA.

Funnel plots alone are insufficient for NMA. Consider Comparison-adjusted funnel plots, Small-study effects analysis

**********

7. PLOS authors have the option to publish the peer review history of their article (what does this mean?). If published, this will include your full peer review and any attached files.

If you choose “no”, your identity will remain anonymous but your review may still be made public.

**Do you want your identity to be public for this peer review?**  For information about this choice, including consent withdrawal, please see our Privacy Policy.

Reviewer #1: **Yes:**  Indu Asanka Jayawardane; Kavinda Madushan Jayasinghe

Reviewer #2: **Yes:**  Michael Sylvester Archibong

**********

[NOTE: If reviewer comments were submitted as an attachment file, they will be attached to this email and accessible via the submission site. Please log into your account, locate the manuscript record, and check for the action link "View Attachments". If this link does not appear, there are no attachment files.]

To ensure your figures meet our technical requirements, please review our figure guidelines: https://journals.plos.org/plosone/s/figures

You may also use PLOS’s free figure tool, NAAS, to help you prepare publication quality figures: https://journals.plos.org/plosone/s/figures#loc-tools-for-figure-preparation.

NAAS will assess whether your figures meet our technical requirements by comparing each figure against our figure specifications.

# Author comment: Author response to Decision Letter 1

**DOI:** 10.1371/journal.pone.0354489.r003

22 Jun 2026

Effect of different oral intake managements during labor on maternal and newborn outcomes: a protocol of systematic review and network meta-analysis

Manuscript ID: PONE-D-25-66860

Response to the Editor

Comment 1: Please ensure that your manuscript meets PLOS ONE's style requirements, including those for file naming. The PLOS ONE style templates can be found at

https://journals.plos.org/plosone/s/file?id=wjVg/PLOSOne_formatting_sample_main_body.pdf and

https://journals.plos.org/plosone/s/file?id=ba62/PLOSOne_formatting_sample_title_authors_affiliations.pdf

Response: Thank you for your helpful comments. We have carefully revised the manuscript to ensure that it complies with the PLOS ONE formatting requirements. Specifically, we have adjusted the title to sentence case format, standardized the author names and affiliations, and revised the manuscript according to the PLOS ONE templates. All formatting issues have been checked and corrected throughout the manuscript. (page1, title page).

Comment 2: Please provide a complete Data Availability Statement in the submission form, ensuring you include all necessary access information or a reason for why you are unable to make your data freely accessible. If your research concerns only data provided within your submission, please write "All data are in the manuscript and/or supporting information files" as your Data Availability Statement.

Response: Thank you for your comment. As this study is a protocol for a systematic review and network meta-analysis, no datasets were generated or analyzed. We have provided a complete Data Availability Statement in both the manuscript and the submission system, clarifying that all data will be obtained from published studies. (page 2, Data Availability Statement section)

Comment 3�When completing the data availability statement of the submission form, you indicated that you will make your data available on acceptance. We strongly recommend all authors decide on a data sharing plan before acceptance, as the process can be lengthy and hold up publication timelines. Please note that, though access restrictions are acceptable now, your entire data will need to be made freely accessible if your manuscript is accepted for publication. This policy applies to all data except where public deposition would breach compliance with the protocol approved by your research ethics board. If you are unable to adhere to our open data policy, please kindly revise your statement to explain your reasoning and we will seek the editor's input on an exemption. Please be assured that, once you have provided your new statement, the assessment of your exemption will not hold up the peer review process.

Response: Thank you for your comment. We have revised the data availability statement in the submission form accordingly. As this study is a protocol for a systematic review and network meta-analysis, no datasets were generated or analyzed at this stage. We have therefore clarified that all data will be obtained from previously published studies, and have removed the statement indicating that data would be made available upon acceptance. (page 2, Data Availability Statement section).

Comment 4�Please include captions for your Supporting Information files at the end of your manuscript, and update any in-text citations to match accordingly. Please see our Supporting Information guidelines for more information: http://journals.plos.org/plosone/s/supporting-information.

Response: We have added captions for all Supporting Information files at the end of the manuscript and updated the in-text citations to ensure consistency with the journal’s requirements. (page 9, Supporting information section).

Comment 5�If the reviewer comments include a recommendation to cite specific previously published works, please review and evaluate these publications to determine whether they are relevant and should be cited. There is no requirement to cite these works unless the editor has indicated otherwise.

Response�Thank you for your guidance and will take it into consideration.

Response to the reviewer 1

Comment 1�The proposal needs language refinements throughout the protocol for clarity.

Response: Thank you for this valuable comment. We have carefully revised the language throughout the manuscript to improve clarity, readability, and academic expression. Specifically, grammatical structures, sentence flow, and terminology have been thoroughly refined. All revisions have been highlighted in the revised manuscript for ease of review.

Comment 2�The abstract mentions "any other" secondary outcomes. Secondary outcomes need to be clearly stated and pre-specified to avoid bias.

Response: Thank you for this comment. We have clearly predefined and fully listed all secondary outcomes in both the abstract and main text. All vague expressions have been removed, and no additional outcomes will be added. This revision improves transparency and consistency with the protocol. (page 4, Outcomes section).

Comment 3�Eligibility criteria. How can you include unpublished RCTs?

Response: Thank you for this important comment. We have revised the eligibility criteria to clarify that only full-text published randomized controlled trials will be included. Unpublished studies, conference abstracts, and ongoing trials without full texts will be excluded. This clarification improves the feasibility and consistency of the study design. (page 4, Types of studies section).

Comment 4�Please specify how to assess "vomiting rate"

Response: Thank you for this comment. Vomiting rate has been clarified in the secondary outcomes as reported in the original studies, and the revision has been marked in red. (page 4, Secondary Outcomes section).

Comment 5�Outcomes of ‘sensations of thirst and hunger’ and ‘maternal satisfaction’ seems overlapping.

Response: Thank you for this comment. We agree that these two outcomes may appear conceptually similar. We have clarified that “thirst and hunger sensations” refer to maternal subjective physical discomfort during labor, whereas “maternal satisfaction” reflects the overall evaluation of the birth experience. Both outcomes are retained as they measure different constructs. (page 4, Secondary Outcomes section).

Comment 6�Protocol emphasizes the use of different statistical packages: RevMan, R software, STATA…. Explicitly state how, when and where those will be used.

Response: Thank you for this comment. We have clarified the specific roles of the statistical software packages used in this review in the Data synthesis section. RevMan 5.4 will be used for pairwise meta-analysis and data management, R software for Bayesian network meta-analysis, and STATA 18.0 for network plotting and assessment of publication bias. The corresponding revisions have been highlighted in red. (pages 7-8, Data synthesis section, Network meta-analysis section and Assessment of reporting biases section).

Response to the reviewer 2

Comment 1: Insufficient detail on network meta-analysis methodology: Important missing details include: Model specification (fixed vs random for NMA — only random mentioned generically),Prior distributions (Bayesian analysis requires specification), Convergence diagnostics (e.g., Gelman-Rubin, trace plots), Number of iterations / burn-in, Assessment of transitivity assumption, Assessment of consistency/inconsistency, node-splitting, Design-by-treatment interaction model, Handling of multi-arm trials, Ranking uncertainty interpretation. These are essential for reproducibility and credibility.

Response: Thank you for this valuable comment. We have added a new Network meta-analysis section in the Data synthesis part of the revised manuscript to provide more detailed methodological descriptions as suggested. The corresponding revisions have been highlighted in red. (page 8, Network meta-analysis section).

Comment 2: Intervention classification is unclear: The intervention category “oral intake management” is extremely broad. Examples listed include: Fasting, Clear fluids, Carbohydrate drinks, High-energy liquids, Individualized regimens, Unrestricted intake. Without predefined grouping rules, there is high risk of Clinical heterogeneity. The protocol should specify Planned intervention nodes, Criteria for merging/splitting nodes, Handling of mixed interventions (e.g., fluids + solids).

Response: Thank you for this valuable comment. We agree that the original description of oral intake interventions was too broad and may lead to ambiguity in network meta-analysis. In the revised manuscript, we have specified predefined intervention nodes, clarified the criteria for grouping interventions, and described how mixed interventions will be handled. These revisions have been highlighted in red in the manuscript. (page 7, Intervention classification section).

Comment 3: Outcome selection rationale is unclear. Primary outcomes include delivery mode and neonatal hypoglycemia. Some outcomes may be rare (e.g., Mendelson’s syndrome).Maternal satisfaction is listed as secondary but is highly relevant. The authors should Provide clinical reasoning, Define time points clearly, Specify measurement definitions (e.g., PPH threshold)

Response: Thank you for this comment. We have revised the Outcome Measures section to provide the clinical rationale for outcome selection and to clarify key outcome definitions. We have also specified that outcome definitions and assessment time points will be extracted as reported in the original studies. In addition, we clarified the inclusion of rare but clinically important adverse events, such as Mendelson’s syndrome. All revisions have been highlighted in red. (page 4, Outcome measures section).

Comment 4: Pairwise analysis plan is confusing. The protocol states: A pairwise Bayesian meta-analysis followed by a network meta-analysis.Typically, Pairwise meta-analysis is frequentist NMA can be Bayesian or frequentist, Clarification is needed.

Response: Thank you for this helpful comment. We have clarified the statistical framework used for pairwise and network meta-analyses. Pairwise meta-analyses are now described separately in the Data Synthesis section and will be conducted in RevMan 5.4 using a random-effects model. The Bayesian random-effects network meta-analysis is described in a separate Network Meta-analysis section. The relevant revisions have been highlighted in red. (pages 7-8, Data synthesis section and Network meta-analysis section).

Comment 5: Software inconsistency. The manuscript mentions RevMan 5.4, R software, STATA 18. This creates confusion regarding Primary analytic platform and Reproducibility pipeline. A clearer analytic workflow is needed.

Response: Thank you for this comment. We have clarified the analytical workflow throughout the manuscript. RevMan 5.4 will be used for data management and pairwise meta-analyses, R software for Bayesian network meta-analysis, and STATA 18.0 for publication bias assessment. These functions are now specified in the corresponding sections and highlighted in red. (pages 7-8, Data synthesis section, Network meta-analysis section and Assessment of reporting biases section).

Comment 6: Risk of bias synthesis not describe. While RoB2 is planned, the protocol does not state how bias will influence analysis, Sensitivity analyses, Exclusion of high-risk studies, and Meta-regression. This should be specified.

Response: Thank you for this comment. We have revised the Risk of Bias Assessment section to clarify how risk of bias will be incorporated into the review. Sensitivity analyses excluding studies at high risk of bias may be conducted where appropriate, and the risk of bias assessment will be considered when interpreting the findings and assessing the certainty of evidence. The relevant revisions have been highlighted in red. (pages 6-7, Risk of bias assessment section).

Response to Reviewer – Moderate concern

Comment 1: Search strategy may miss relevant studies. The PubMed search appears limited in synonyms for labor nutrition, Does not clearly include anesthesia-related terms that may capture relevant trials. A full reproducible search strategy for all databases should be included in supplementary materials

Response: Thank you for this comment. We have clarified that a comprehensive literature search will be conducted across multiple databases using both Medical Subject Headings (MeSH) terms and free-text keywords. Equivalent search strategies are applied across all databases to ensure consistency and comprehensiveness. The detailed PubMed search strategy is provided in Table S2. The manuscript has been revised accordingly. (page 5, Information sources and search strategy section).

Comment 2: Heterogeneity description is simplistic. Statement that “non-overlapping confidence intervals indicate heterogeneity” is incorrect or oversimplified. Heterogeneity assessment should rely on:I², τ² and Clinical judgment.

Response: Thank you for this comment. We have revised the Assessment of Heterogeneity section by removing the statement regarding non-overlapping confidence intervals and clarifying that heterogeneity will be assessed using clinical and methodological judgment together with statistical measures, including the I² statistic and between-study variance (τ²), where appropriate. The relevant revisions have been highlighted in red. (page 8, Assessment of Heterogeneity section).

Comment 3: Reporting bias assessment in NMA. Funnel plots alone are insufficient for NMA. Consider Comparison-adjusted funnel plots, Small-study effects analysis.

Response: Thank you for this comment. We have revised the Assessment of Reporting Biases section to clarify that, where sufficient studies are available, both funnel plots and comparison-adjusted funnel plots will be used to assess potential publication bias and small-study effects in the network meta-analysis. The relevant revisions have been highlighted in red. (page 8, Assessment of Reporting Biases section).

Attachment Submitted filename: PLOS_One__Response_to_Reviewers_auresp_1.docx

# Peer review: Decision Letter 1

**DOI:** 10.1371/journal.pone.0354489.r004

**Authors:** Ricardo Ney Oliveira Cobucci

8 Jul 2026

Effects of different oral intake management during labor on maternal and neonatal outcomes: A protocol for a systematic review and network meta-analysis.

PONE-D-25-66860R1

Dear Dr. Chen,

We’re pleased to inform you that your manuscript has been judged scientifically suitable for publication and will be formally accepted for publication once it meets all outstanding technical requirements.

Within one week, you’ll receive an e-mail detailing the required amendments. When these have been addressed, you’ll receive a formal acceptance letter and your manuscript will be scheduled for publication.

An invoice will be generated when your article is formally accepted. Please note, if your institution has a publishing partnership with PLOS and your article meets the relevant criteria, all or part of your publication costs will be covered. Please make sure your user information is up-to-date by logging into Editorial Manager at Editorial Manager® and clicking the ‘Update My Information' link at the top of the page. For questions related to billing, please contact billing support.

If your institution or institutions have a press office, please notify them about your upcoming paper to help maximize its impact. If they’ll be preparing press materials, please inform our press team as soon as possible -- no later than 48 hours after receiving the formal acceptance. Your manuscript will remain under strict press embargo until 2 pm Eastern Time on the date of publication. For more information, please contact onepress@plos.org.

Kind regards,

Ricardo Ney Oliveira Cobucci, Ph.D

Academic Editor

PLOS One

Additional Editor Comments (optional):

Reviewers' comments:

Reviewer's Responses to Questions

**Comments to the Author**

1. Does the manuscript provide a valid rationale for the proposed study, with clearly identified and justified research questions?

The research question outlined is expected to address a valid academic problem or topic and contribute to the base of knowledge in the field.

Reviewer #1: Yes

**********

2. Is the protocol technically sound and planned in a manner that will lead to a meaningful outcome and allow testing the stated hypotheses?

The manuscript should describe the methods in sufficient detail to prevent undisclosed flexibility in the experimental procedure or analysis pipeline, including sufficient outcome-neutral conditions (e.g. necessary controls, absence of floor or ceiling effects) to test the proposed hypotheses and a statistical power analysis where applicable. As there may be aspects of the methodology and analysis which can only be refined once the work is undertaken, authors should outline potential assumptions and explicitly describe what aspects of the proposed analyses, if any, are exploratory.

Reviewer #1: Yes

**********

3. Is the methodology feasible and described in sufficient detail to allow the work to be replicable?

Descriptions of methods and materials in the protocol should be reported in sufficient detail for another researcher to reproduce all experiments and analyses. The protocol should describe the appropriate controls, sample size calculations, and replication needed to ensure that the data are robust and reproducible.

Reviewer #1: Yes

**********

4. Have the authors described where all data underlying the findings will be made available when the study is complete?

The PLOS Data policy requires authors to make all data underlying the findings described in their manuscript fully available without restriction, with rare exception, at the time of publication. The data should be provided as part of the manuscript or its supporting information, or deposited to a public repository. For example, in addition to summary statistics, the data points behind means, medians and variance measures should be available. If there are restrictions on publicly sharing data—e.g. participant privacy or use of data from a third party—those must be specified.

Reviewer #1: Yes

**********

5. Is the manuscript presented in an intelligible fashion and written in standard English?

PLOS ONE does not copyedit accepted manuscripts, so the language in submitted articles must be clear, correct, and unambiguous. Any typographical or grammatical errors should be corrected at revision, so please note any specific errors here.

Reviewer #1: Yes

**********

6. Review Comments to the Author

Please use the space provided to explain your answers to the questions above and, if applicable, provide comments about issues authors must address before this protocol can be accepted for publication. You may also include additional comments for the author, including concerns about research or publication ethics.

You may also provide optional suggestions and comments to authors that they might find helpful in planning their study.

(Please upload your review as an attachment if it exceeds 20,000 characters)

Reviewer #1: I congratulate the authors on getting the review done. I read the current manuscript with interest. Previous reviewer comments have been addressed satisfactorily. I have no further comments to make.

**********

7. PLOS authors have the option to publish the peer review history of their article (what does this mean?). If published, this will include your full peer review and any attached files.

If you choose “no”, your identity will remain anonymous but your review may still be made public.

**Do you want your identity to be public for this peer review?**  For information about this choice, including consent withdrawal, please see our Privacy Policy.

Reviewer #1: **Yes:**  Indu Asanka Jayawardane

**********

# Editor report: Acceptance letter

**DOI:** 10.1371/journal.pone.0354489.r005

**Authors:** Ricardo Ney Oliveira Cobucci

PONE-D-25-66860R1

PLOS One

Dear Dr. Chen,

I'm pleased to inform you that your manuscript has been deemed suitable for publication in PLOS One. Congratulations! Your manuscript is now being handed over to our production team.

At this stage, our production department will prepare your paper for publication. This includes ensuring the following:

* All references, tables, and figures are properly cited

* All relevant supporting information is included in the manuscript submission,

* There are no issues that prevent the paper from being properly typeset

You will receive further instructions from the production team, including instructions on how to review your proof when it is ready. Please keep in mind that we are working through a large volume of accepted articles, so please give us a few days to review your paper and let you know the next and final steps.

Lastly, if your institution or institutions have a press office, please let them know about your upcoming paper now to help maximize its impact. If they'll be preparing press materials, please inform our press team within the next 48 hours. Your manuscript will remain under strict press embargo until 2 pm Eastern Time on the date of publication. For more information, please contact onepress@plos.org.

You will receive an invoice from PLOS for your publication fee after your manuscript has reached the completed accept phase. If you receive an email requesting payment before acceptance or for any other service, this may be a phishing scheme. Learn how to identify phishing emails and protect your accounts at https://explore.plos.org/phishing.

If we can help with anything else, please email us at customercare@plos.org.

Thank you for submitting your work to PLOS ONE and supporting open access.

Kind regards,

PLOS ONE Editorial Office Staff

on behalf of

PROFESSOR Ricardo Ney Oliveira Cobucci

Academic Editor

PLOS One
